# Expression and prognosis of inducible T‐cell co‐stimulator and its ligand in Chinese stage I–III lung adenocarcinoma patients

**DOI:** 10.1002/ame2.12355

**Published:** 2023-10-18

**Authors:** Xiao‐Kai Zhan, Xi‐Kun Liu, Sen Zhang, Hong Chen

**Affiliations:** ^1^ Division of Oncology and Hematology Beijing Chao‐Yang Hospital, Capital Medical University Beijing P.R. China; ^2^ State Key Laboratory of Bioactive Substances and Functions of Natural Medicines, Institute of Materia Medica Chinese Academy of Medical Sciences and Peking Union Medical College Beijing P.R. China; ^3^ Pathology Department Beijing Chao‐Yang Hospital, Capital Medical University Beijing P.R. China

**Keywords:** Chinese patients, ICOS, ICOSL, lung adenocarcinoma, prognostic factor

## Abstract

**Background:**

Immunotherapy has become the fastest‐adopting treatment paradigm for lung cancer with improved survival. By binding with its ligand (inducible T‐cell co‐stimulator and its ligand [ICOSL]), an inducible T‐cell co‐stimulator (ICOS) could contribute to reversing immunosuppression and improving immune response and thus be a potential target for cancer immunotherapy.

**Methods:**

We selected 54 formalin‐fixed, paraffin‐embedded tumor tissues from cases with stage I–III lung adenocarcinoma cancer. Immunohistochemical expression of ICOS and ICOSL was evaluated. The correlation with clinical parameters in Chinese patients was also compared with TCGA results.

**Results:**

The positive rates of ICOS and ICOSL were 68% and 81.5%, respectively, in lung tumor tissues. Of these, 9 cases had a low expression of ICOS, and 22 cases had a high expression of ICOS; ICOSL expression was low in 20 cases and high in 24 cases. According to the International Association for the Study of Lung Cancer (8th edition), phase I lesions were detected in 21 cases, phase II lesions in 15 cases, and phase III lesions in 18 cases. The median survival time of all patients was 44.5 months, and the median disease‐free survival was 32 months. Univariate analysis showed that the factors significantly associated with overall survival were tumor size, regional lymph node involvement, stage, and expression level of ICOS/ICOSL. Survival analysis using log‐rank test indicated that the lower ICOS+ cell infiltration may predict poor prognosis, whereas lower ICOSL protein expression may be associated with better prognosis, but ICOSL data need further validation in larger samples due to inconsistency in TCGA mRNA prediction.

**Conclusion:**

ICOS/ICOSL might be associated with prognosis of lung cancer, and ICOS and its ligand may be potential therapeutic targets in non‐small cell lung cancer.

## Graphical abstract

This study included the data of patients who were diagnosed with adenocarcinoma by pathology after initial treatment and radical operation in Beijing Chaoyang Hospital from October 2016 to January 2018. The relevant medical records of patients were collected and sorted out according to the demographic, clinical, and pathological features, curative treatment effect evaluation, and long‐term survival after treatment. Immunohistochemical analyses were performed according to methods published by Budwit‐Novotny et al. The expression of T‐cell co‐stimulator and inducible T‐cell co‐stimulator and its ligand were divided to negative, low and high.

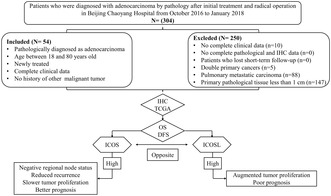

## 1 INTRODUCTION

Lung cancer is a serious threat to human life globally and the leading cause of cancer deaths.^1^ About 85% of cases are non‐small cell lung cancer (NSCLC), of which the main pathological type is lung adenocarcinoma (LUAD).^2^, ^3^ Although surgeries and chemotherapy have provided modest survival gains, patients with recurrent and advanced lung cancer often progress and still have very poor prognosis.^4^ In recent years, with the breakthrough of immune checkpoint inhibitor therapy in recurrent and refractory lung cancer,^5^, ^6^ more attention has been paid to understand in depth the tumor immune system.

The B7‐CD28 immunoglobulin family consists of the most extensive T‐cell co‐stimulatory molecules. Of these, inhibitory programmed death molecule‐1 and cytotoxic T‐lymphocyte‐associated antigen‐4 have become hot spots in cancer treatment.^7^ Besides these, inducible T‐cell co‐stimulator (ICOS), belonging to the B7‐CD28 family, provides identification signals to activate T cells.^8^ By binding with its only ligand (inducible T‐cell co‐stimulator and its ligand [ICOSL]), it plays an important role in regulating T‐cell activation and differentiation, participating in T‐/B‐cell coordination, and promoting cytotoxic tell‐lymphocyte response, and it can resist immunosuppression and improve immune response efficiency.^9^ At present, the antitumor effect of the ICOS/ICOSL pathway has been recognized. Emerging literature reports that ICOS or ICOSL is dysregulated in various cancer types and contributes to tumor immune escape.^10^ ICOS/ICOSL expressed in regulatory T‐cells (Tregs) within the tumor microenvironment largely influences T‐cell‐mediated tumor immune tolerance. For example, ICOS+ tumor‐infiltrating lymphocytes (TIL) remarkably increased in advanced gastric cancer and represent poor prognosis^11^; ICIs combined with ICOS agonist GSK3359609 could exert better antitumor effects by enhancing T‐cell cytotoxicity in head and neck squamous cell carcinoma (clinical trial information: NCT04128696). However, the roles of ICOS/ICOSL in lung cancer are not very clear.

This study explored ICOS and ICOSL expression and their correlation with clinical outcomes in human NSCLC using TCGA data mining; also immunohistochemistry analysis was performed using freshly resected tumor tissue from selected lung cancer patients. We found that ICOS‐ICOSL axis activation may influence tumor stages, overall survival (OS), and disease‐free survival (DFS). Ours is the first comprehensive exploratory study of the expression and prognosis of ICOS and ICOSL in lung cancer in Chinese patients.

## 2 PATIENTS AND METHODS

### 2.1 Patients and samples

This study included the data of patients who were diagnosed with adenocarcinoma by pathology after initial treatment and radical operation in Beijing Chaoyang Hospital from October 2016 to January 2018. The procedure was as follows: the relevant medical records of patients were consulted; details of the demographic, clinical, and pathological features; curative treatment effect evaluation; and long‐term survival after treatment were collected and sorted out. All the patients in this group were routinely examined before operation, including blood test, liver and kidney function, and blood coagulation. Based on the results of related auxiliary examinations such as computed tomography (CT) of chest and abdomen or positron emission tomography CT, the primary site was determined, and patients with metastasis of other sites were excluded. All patients did not receive any treatment before operation and signed an informed consent form in the thoracic surgery department of our hospital for surgical treatment. After the operation, the pathological specimens were collected, which were confirmed as adenocarcinoma by three pathologists in our hospital, and the double primary cancers were excluded by multidisciplinary consultation. All patients' tissue specimens were pathologically diagnosed based on the classification of lung tumors published by the World Health Organization (WHO) in 2008. Based on the patients' physical examination before treatment, physical condition score (based on the behavior status of the Eastern Cooperative Oncology Group, PS) and staging (based on the eighth edition of the International Association for Research on Lung Cancer)^12^ were mainly recorded. This study was approved by the Ethics Committee of Chaoyang Hospital (approval number: 2021‐5‐28‐5), and documents for consent to participate were obtained from all patients.

Thus, patients were included in the study if they (1) were aged 18–80 years, (2) were pathologically diagnosed as adenocarcinoma according to the classification of lung tumors published by the WHO in 2008, (3) received routine examinations before operation, (4) had complete clinical data, and (5) had complete pathological and immunohistochemical data. Patients were excluded if they (1) had double primary cancer, (2) had pulmonary metastatic carcinoma, (3) received any treatment before operation, (4) lost short‐term follow‐up after visiting the hospital, and (5) had a history of other malignant tumors.

### 2.2 Immunohistochemistry

Immunohistochemical analyses were performed based on the methods published by Budwit‐Budwit‐Novotny et al.^13^ Briefly, the tissue sections were dewaxed with xylene and hydrated with ethanol of different concentrations. After antigen retrieval using citrate buffer, they were incubated with primary antibodies against ICOS and ICOSL. After incubation, the sections were incubated with secondary antibodies, stained with a DAB Quanto Kit (Golden Bridge Biological Technology), and then counterstained with hematoxylin. After the sheet was dried, it was sealed with a cover glass, and images were obtained for analysis using an inverted microscope.

**FIGURE 1:**
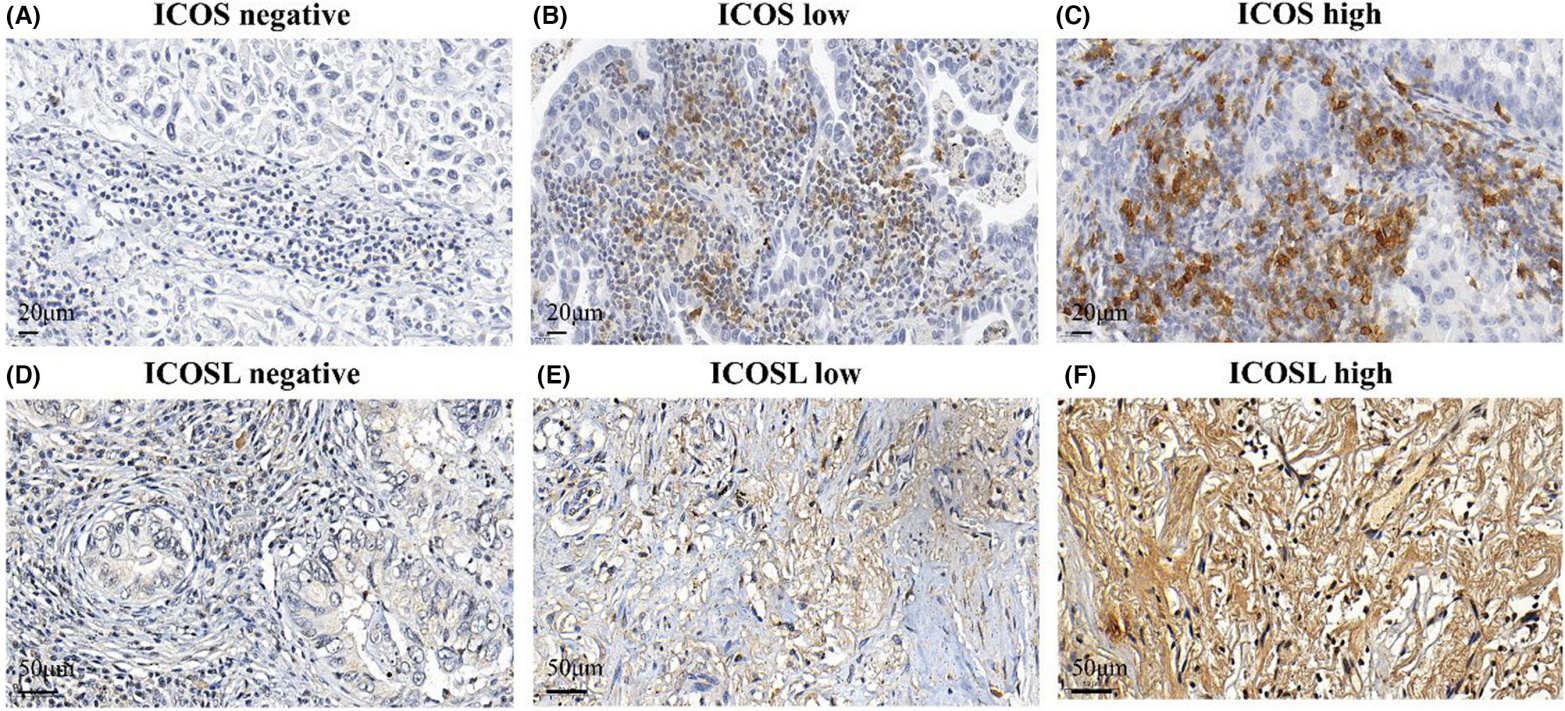
Representative images of immunohistochemical analyses of ICOS and ICOSL expressions. (A) Negative ICOS expression, (B) low ICOS expression, (C) high ICOS expression, (D) negative ICOSL expression, (E) low ICOSL expression, and (F) high ICOSL expression. ICOS, inducible T‐cell co‐stimulator; ICOSL, inducible T‐cell co‐stimulator and its ligand.

The expression of ICOS and ICOSL was divided into negative, low, and high (Figure 1A–F). And the expression intensity criteria of ICOS and ICOSL were as follows: Image‐Pro software was used to measure the staining area, and the percentage of each HPF‐positive area in the whole section was defined as positive area. Extent was classified as 0, ≤5%; 1, 6 to ≤15%; 2, 16 to ≤30%; 3, 31 to ≤50%; 4, 50%–75%; and 5, >75%. Intensity of staining was classified as 0, negative; 1, weak; 2, moderate; and 3, strong based on professional experience by two pathologists. The cutoff value of the expression group was determined by the degree × intensity staining level and the final score. Then, the protein expression was divided into three categories: 0–3, “negative”; 4–6, “low”; and ≥6, high expression.

### 2.3 Treatment and follow‐up

Routine chemotherapy is not recommended for stage I patients after operation. For patients with postoperative high‐risk factors, adjuvant chemotherapy was used for stages II and IIIA after complete resection of lesions. Postoperative adjuvant radiotherapy was conducted for peri‐pleural lesions or lymph node lesions or larger single lymph nodes. For local recurrence, reoperation or radiotherapy was used, followed by chemotherapy or targeted therapy, whereas for metastasis, chemotherapy or targeted therapy and immunotherapy were used. Treatment was selected in accordance with NCCN guidelines.

Patients were followed up until July 1, 2021, by telephone or regular outpatient reexamination, and the main form of follow‐up was outpatient postoperative reexamination once every 3 months. If necessary, the patients were examined based on the symptoms, such as chest and abdomen imaging examination, and a head magnetic resonance imaging or bone scan was added if necessary. At each follow‐up, the patients' survival and disease progression, as well as the causes of recurrence or death, were obtained. No single patient was lost to follow‐up.

### 2.4 Research endpoint

The primary endpoint of the study was OS, and the secondary endpoint was DFS. OS is defined as the time from diagnosis to death or the last visit where recorded. DFS is defined as the time from the operation to the recurrence of the disease or the death of the patient due to the progression of the disease. Records of patients were not available for the last follow‐up or had no outcome events were deemed invalid.

### 2.5 Statistical analysis

SPSS 24.0 (SPSS Inc.) software was used for statistical analysis. Kaplan–Meier method was used to draw the survival curve.^14^ The rank‐sum test and *χ* ^2^ test were used for intergroup comparison. Comparison of survival rates among groups and univariate analysis were performed using log‐rank test. *p* < 0.05 was considered as statistically significant.

### 2.6 Data mining of TCGA cohort in OncoLnc database

The corresponding patient follow‐up data on LUAD (Figure 3, *n* = 492) were acquired from OncoLnc (www.oncolnc.org). TCGA expression data (ICOS and ICOSL) quantified as RSEM (RNA‐Seq by expectation maximization) were logarithmically transformed using the UALCAN website (http://ualcan.path.uab.edu/analysis.html). The analyzed TCGA data of LUAD about the relationship between the expression level of ICOS and its ligand and OS and DFS were downloaded from the GEPIA2 database^15^ (Figure 4; http://gepia2.cancer‐pku.cn/#correlation).

## 3 RESULTS

### 3.1 Clinical and pathological characteristics of patients

Fifty‐four patients who underwent radical surgical resection from October 2016 to July 2020 were recruited for the present study. In this study, most patients are elderly, and the median age of the population was 61 years; the ratio of males to females was 1:1. Of these patients, 26 had a history of smoking, approximately accounting for half of the patients. According to the International Association for the Study of Lung Cancer (8th edition), 21 cases were classified as stage I, 15 as stage II, and 18 as stage III. The median tumor size was 2.25 cm (ranged from 1.1 to 6.7 cm), and 16 samples had tumor size more than 3 cm. Twenty‐one cases had regional lymph node metastasis. ICOS was found to be positive in 58% (*n* = 31 of 54) of all lung cancer samples, and ICOS was categorized as negative in 42% (*n* = 23 of 54), low in 17% (*n* = 9 of 54), and high in 41% (*n* = 22 of 54). Similarly, ICOSL expression was detected in 81.5% (*n* = 44 of 54) of the total tumor tissues. ICOSL was scored as negative in 19% (*n* = 10 of 54), low in 37% (*n* = 20 of 54), and high in 44% (*n* = 24 of 54). After follow‐up, 45 cases survived, and the median OS was 44.5 months (ranged from 10 to 57 months); 20 relapsed, and the median DFS was 32 months (ranged from 10 to 57 months). Baseline patient demographics and clinical characteristics are presented in Table 1.

**TABLE 1:** Clinical and pathological characteristics of patients.

Parameter	Subjects (*N* = 54)	
Age (year)	Median (range)	61 (38–77)
	≤60	22
	>60	32
Gender	Male	27 (50%)
	Female	27 (50%)
Smoking history	Yes	26 (48%)
	No	28 (52%)
Stage	I	21 (39%)
	II	15 (28%)
	III	18 (33%)
ICOS	Negative	23 (42%)
	Low	9 (17%)
	High	22 (41%)
ICOSL	Negative	10 (19%)
	Low	20 (37%)
	High	24 (44%)
Median tumor size (cm)	≤3	38
	>3	16
Regional nodes	Negative	33
	Positive	21
Survival months	Range	44.5 (10–57)
	Yes	45
	No	9
DFS months (range)		32 (10–57)
Recurrence	Yes	20
	No	34

Abbreviations: DFS, disease‐free survival; ICOS, inducible T‐cell co‐stimulator; ICOSL, inducible T‐cell co‐stimulator and its ligand.

### 3.2 Immunohistochemical analysis of overall ICOS/ICOSL expression in lung cancer

This study investigated the distribution of ICOS and ICOSL expression in different groups, including gender, age, smoking history, tumor size, regional node metastasis, different stages, recurrence, and survival. The protein levels of ICOS and ICOSL were categorized as negative, high, and low using the IHC system. The different categories for each group are presented in Tables 2 and 3. After pairwise comparisons were performed, there was no significant difference among the four groups (gender, age, smoking history, and survival status) (*p* > 0.05). On the contrary, the expression of ICOS in small‐sized tumors (≤3 cm) was significantly higher than that in bigger‐sized tumors (>3 cm) (*p* = 0.01). Additionally, there was a significant difference in regional lymph node metastasis, different stages, and recurrence states. It seemed that the higher protein level of ICOS was mostly found in cases with smaller tumors, negative regional node metastasis, no recurrence, and lower stage. Moreover, ICOS protein levels were significantly associated with different stages, lymph node metastasis, tumor size, and recurrence status (Table 2). Nevertheless, the expression of ICOSL was not correlated with age, gender, smoking history, stage, lymph node involvement, and recurrence status (*p* > 0.05). Its expression was associated with different stages and tumor size (Table 3).

**TABLE 2:** ICOS expression and clinical characteristics of patients.

	ICOS			*χ* ^2^	*p*‐Value
	Negative (*n* = 23)	Low (*n* = 9)	High (*n* = 22)		
Gender					
Male	14	6	10	1.62	>0.05
Female	9	3	12		
Age (year)					
≤60	10	5	7	0.43	>0.05
>60	13	4	15		
Smoking history					
No	10	5	11	0.43	>0.05
Yes	13	4	11		
Tumor size (cm)					
≤3	10	7	21	14.85	<0.01
>3	13	2	1		
Regional node status					
Negative	9	3	21	18.52	<0.01
Positive	14	6	1		
Recurrence status					
No	7	7	20	18.65	<0.01
Yes	16	2	2		
Survival					
No	7	1	1	5.67	>0.05
Yes	16	8	21		
Staging					
I	0	0	21	68.3	<0.01
II	6	8	0		
III	17	1	1		

Abbreviation: ICOS, inducible T‐cell co‐stimulator.

**TABLE 3:** ICOSL expression and clinical characteristics of patients.

	ICOSL			*χ* ^2^	*p‐*Value
	Negative (*n* = 10)	Low (*n* = 20)	High (*n* = 24)		
Gender					
Male	4	12	11	1.37	>0.05
Female	6	8	13		
Age (year)					
≤60	5	8	15	0.46	>0.05
>60	5	12	9		
Smoking history					
No	5	8	13	0.89	>0.05
Yes	5	12	11		
Tumor size (cm)					
≤3	9	19	10	17.15	<0.01
>3	1	1	14		
Regional node status					
Negative	8	12	13	2.00	>0.05
Positive	2	8	11		
Recurrence status					
No	6	15	13	2.08	>0.05
Yes	4	5	11		
Survival					
No	1	4	4	0.48	>0.05
Yes	9	16	20		
Staging					
I	6	12	3	15.98	<0.01
II	3	5	7		
III	1	3	14		

Abbreviation: ICOSL, inducible T‐cell co‐stimulator and its ligand.

### 3.3 Treatment and follow‐up

At the time of the last follow‐up, nine patients had died, and of these, six died of relapse, one of myocardial infarction, one of cerebral hemorrhage, and one of respiratory failure caused by severe pulmonary infection. During the follow‐up, it was found that 20 patients had recurrence or metastasis, of whom 12 (60%) had new lesions in the lung, the ipsilateral or contralateral lung. New lesions occurred in the pleura in three cases (15%), the liver in two cases (10%), adrenal gland in one case (5%), the bone in one case (5%), and cervical lymph node in one case (5%).

### 3.4 Survival analysis in correlation with tumor ICOS/ICOSL expression

**FIGURE 2:**
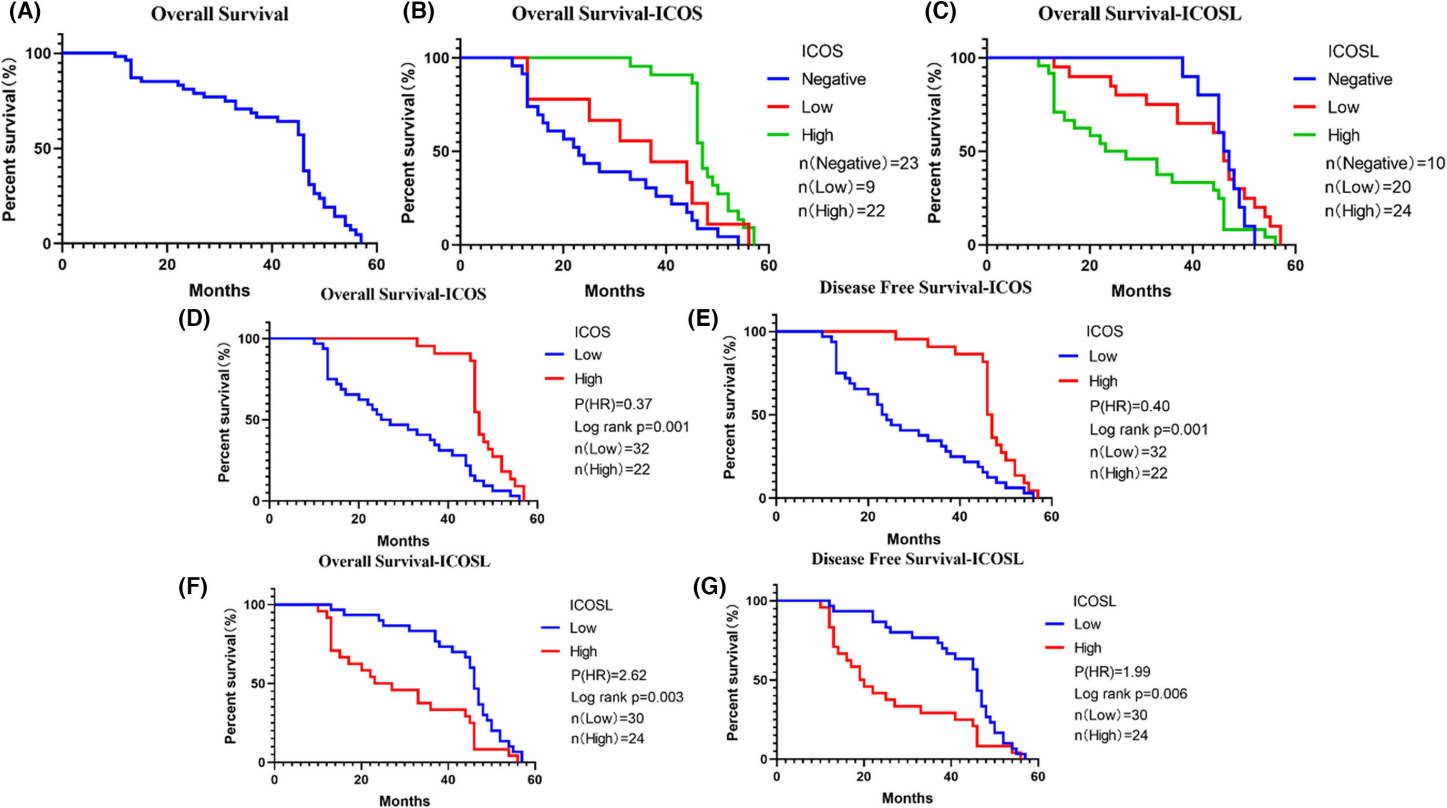
The survival analysis for ICOS and ICOSL expressions by Kaplan–Meier survival curve. (A) The survival curve of 54 NSCLC (non‐small cell lung cancer) patients, (B) expression of ICOS and OS (including immunohistochemistry negative), (C) expression of ICOSL and OS (including immunohistochemistry negative), (D) expression level of ICOS and OS, (E) expression level of ICOS and DFS, (F) expression level of ICOSL and OS, and (G) expression level of ICOSL and DFS. DFS, disease‐free survival; ICOS, inducible T‐cell co‐stimulator; ICOSL, inducible T‐cell co‐stimulator and its ligand; OS, overall survival.

The overall mean and median survival period of the 54 patients were 36.83 and 44.5 months (ranged from 10 to 57 months), respectively (Figure 2A; Table 4). Survival was not associated with gender, age, smoking history, and tumor recurrence (Table 4). However, higher infiltration of ICOS+ cells into lung cancer tumor tissues indicated longer OS period (Figure 2B). The result of the ICOSL expression was contrary, and higher ICOSL expression in tumor tissues predicted poor survival (Figure 2C). We further combined negative staining of ICOS/ICOSL patients and low‐expression patients as the low‐expression group to investigate in depth the association between the expression levels of ICOS/ICOSL and OS and DFS. After analysis, in the low‐expression ICOS group, patients had worse OS (*p* = 0.001, HR = 0.37) and DFS (*p* = 0.001, HR = 0.40) (Figure 2D,E), whereas in the high‐expression ICOSL group, patients had worse OS (HR = 2.62, *p* = 0.003) and DFS (HR = 1.99, *p* = 0.006) compared to the low‐expression group (Figure 2F,G). The results of the univariate analysis implied that the factors such as tumor size, regional node involvement, stage, and protein level of ICOS/ICOSL affected the prognosis of patients (*p* < 0.05). Detailed data are presented in Table 4.

**TABLE 4:** Survival status and tumor ICOS/ICOSL expression.

	Mean OS (months)	Median OS (months)	*p‐*Value
Gender			
Male	45.50	36.5	0.515
Female	44.33	37.0	
Age			
≤60	35.23	40.0	0.985
>60	37.94	45.5	
Smoking history			
No	34.27	37.0	0.425
Yes	39.21	46.0	
Tumor size			
≤3	40.39	46.0	0.013
>3	28.38	24.0	
Regional nodes			
Negative	42.58	47.0	<0.001
Positive	26.83	26	
Recurrence			
No	39.59	46.0	0.063
Yes	32.15	36.0	
ICOS			
Negative	27.17	23.5	<0.001
Low	34.67	37.0	
High	47.82	47.0	
ICOSL			
Negative	48.52	47	0.023
Low	37.27	46.0	
High	22.83	26	
Staging			
I	90	47.5	<0.001
II	83	41	
III	25	18	

Abbreviations: ICOS, inducible T‐cell co‐stimulator; ICOSL, inducible T‐cell co‐stimulator and its ligand; OS, overall survival.

### 3.5 Multiple regression analysis suggested that protein level of ICOS and ICOSL in tumor tissues might predict the prognosis of lung cancer

Given the previous analysis, we found that the expression of ICOS and ICOSL in tumor tissue was significantly correlated with survival, so we utilized a multivariate analysis to evaluate whether ICOS and ICOSL could predict the prognosis of lung cancer. This analysis showed that patients with positive (low or high) ICOS staining mainly had a smaller tumor size (≤3 cm) and longer OS than negative ICOS staining (Tables 2 and 4), whereas patients with negative or low ICOSL staining had a smaller tumor size (≤3 cm) and longer OS (Tables 3 and 4). All these results suggested that ICOS and ICOSL could be used as predictors of lung cancer prognosis.

### 3.6 Comparison with TCGA data

**FIGURE 3:**
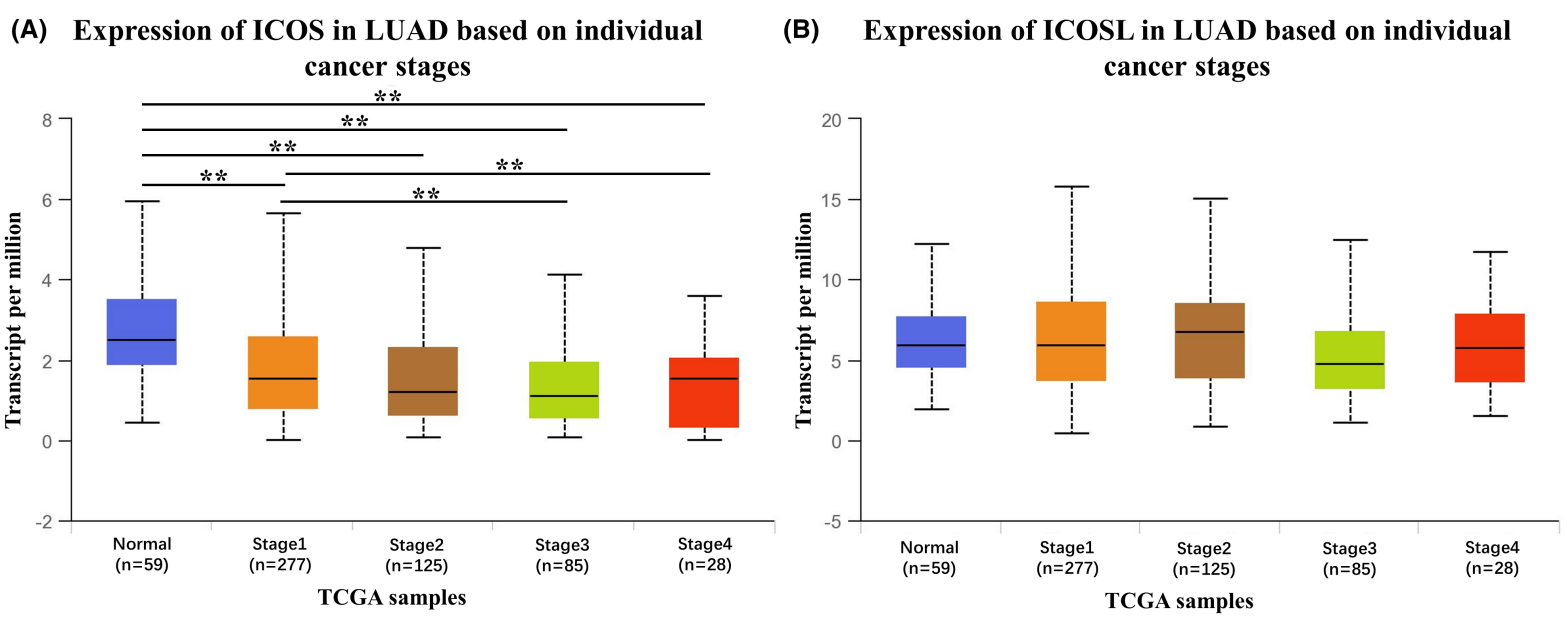
mRNA expression of ICOS and its ligand in different stages of lung adenocarcinoma and normal tissue in TCGA dataset. (A) ICOS expression in different stages and (B) ICOSL expression in different stages. **p < 0.01 using Student's *t*‐test. ICOS, inducible T‐cell co‐stimulator; ICOSL, inducible T‐cell co‐stimulator and its ligand.

Using web analytics TCGA data of UALCAN, the mRNA expression quantity of ICOS and its ligand in different stages of LUAD was compared with that of normal tissue (UALCAN). Figure 3A shows that the ICOS mRNA expression was significantly lower in tumor tissues compared with normal lung tissue (*p* < 0.01), and the mRNA expression in stages III and IV was significantly lower than in that stage 1 (both *p* < 0.01). These results were also consistent with current IHC results, further confirming that ICOS‐positive infiltrated cells predicted better prognosis for lung cancer.

**FIGURE 4:**
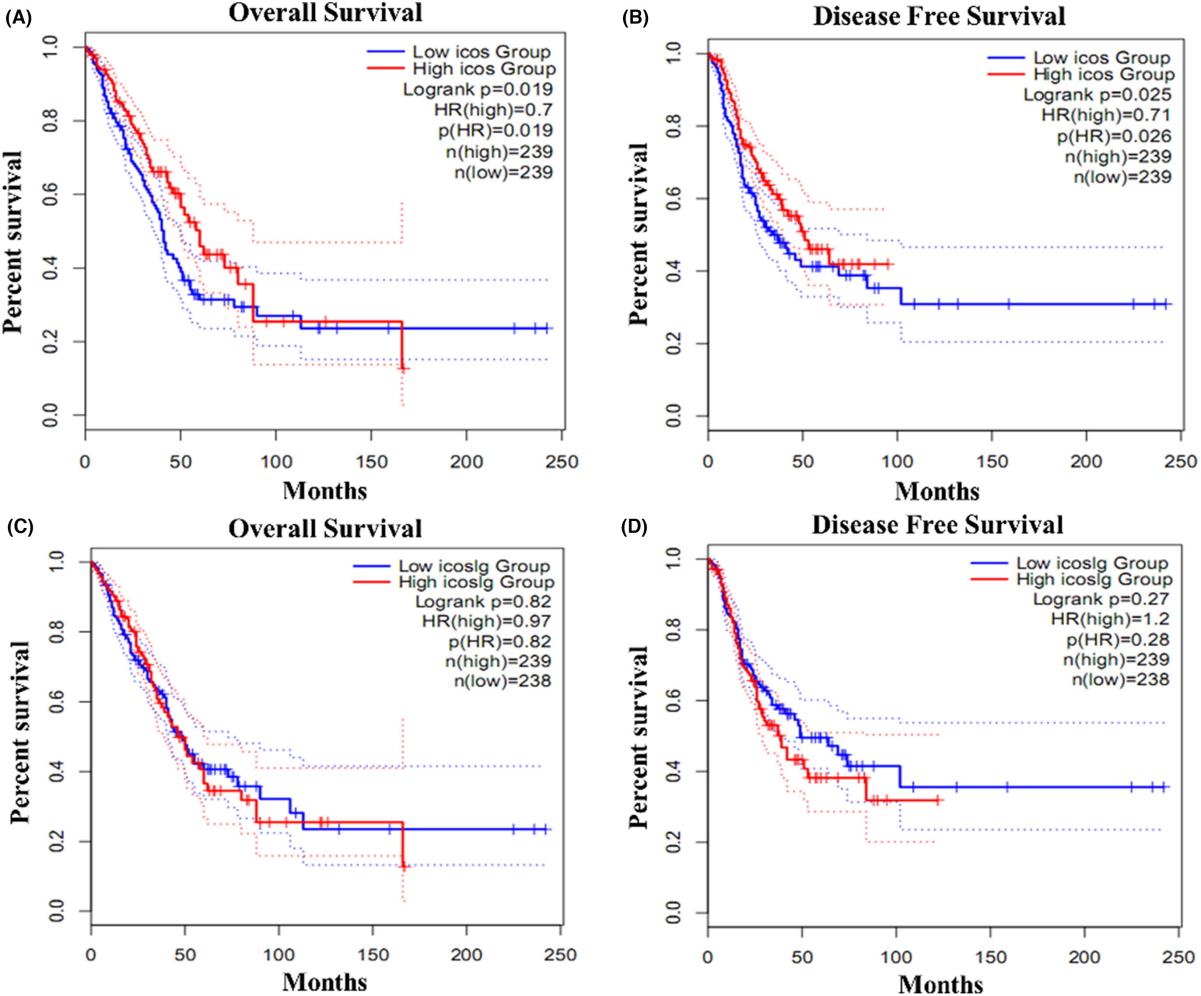
The associations between the mRNA copy number of ICOS and ICOSL genes and survival and DFS outcomes in TCGA dataset. (A) ICOS expression level and OS, (B) ICOS expression level and DFS, (C) ICOSL expression level and OS, and (D) ICOSL expression level and DFS. DFS, disease‐free survival; ICOS, inducible T‐cell co‐stimulator; ICOSL, inducible T‐cell co‐stimulator and its ligand; OS, overall survival.

The data from TCGA reveal the associations between the mRNA copy number of ICOS and ICOSL genes and survival and DFS outcomes. The low‐expression group of ICOS had worse OS and DFS in both sets (*p* = 0.019 and 0.025, HR = 0.70 and 0.71, respectively) compared to the high‐expression group (Figure 4A,B), which was consistent with our immunohistochemistry results. Otherwise, ICOSL was not significantly associated with both OS (*p* = 0.82, HR = 0.82) and DFS (*p* = 0.27, HR = 0.28) (Figure 4C,D) (source from gepia2.cancer database). The mRNA data were inconsistent with IHC results and need further confirmation using larger‐sized samples.

## 4 DISCUSSION

T‐cell activation depends on the stimulation of dual signals: one is the initial antigen recognition and stimulation signal, completed by the combination of antigen peptide‐major histocompatibility complex on the surface of antigen‐presenting cells (APC) and T‐cell receptor on the surface of the T cell, and the other is the co‐stimulation signal, mainly mediated by ICOS.^8^ As a member of the CD28 superfamily, ICOS is mainly expressed on the surface of activated T cells and memory T cells.^16^ Generally, ICOS is weakly expressed in normal nonlymphoid tissues; however, it is over‐expressed in T cells and T‐cell‐differentiating subsets within tumor tissues. It has been found that ICOS mRNA in TIL is highly expressed in human gastric cancer tissues, colorectal cancer tissues, breast cancer, head and neck tumors, and lung cancer.^17^ ICOSL, the unique ligand of ICOS, is a member of the B7 family.^18^ It is expressed on APCs, including B cells, macrophages, dendritic cells, certain endothelial cells, and lung epithelium.^19^, ^20^ By binding to the ligand, the pathway of ICOS/ICOSL provides the signal that is very important for inducing the production of a wide spectrum of cytokines by T‐cell effectors such as CD4 and CD8.^21^, ^22^ In preclinical studies, the pathway of ICOS/ICOSL has been shown to promote antitumor T‐cell responses.^23^ A large body of evidence confirms that activation of the ICOS/ICOSL pathway is involved in the maintenance of T cells in the tumor microenvironment, which is usually associated with a poor prognosis of the patient.^11^ In recent years, expanding studies suggest that ICOS may be a new therapeutic target in cancer,^24^ and there are several clinical trials on ICOS or ICOSL as combination targets with ICIs.

However, a few studies have focused on the relationship between the ICOS/ICOSL expression and prognosis of NSCLC. This article is an exploratory and observational study. We selected all LUAD cases with stages I–III that were pathologically confirmed to achieve N0 complete resection after radical resection. Samples from all patients, the pathological sections of tumor tissues and normal tissue adjacent to cancer cells, were stained with ICOS and ICOSL antibody reagents. To understand the expression and significance of ICOS in LUAD, we observed the OS and DFS of patients without interfering with any treatment of patients. In our study, a relatively high positive expression ratio of ICOS and ICOSL was observed, with an expression rate exceeding 30%. Because receptors and ligands are expressed on different cells, the expression state of receptors and ligands is not consistent. This at least shows that ICOS and its ligand are widely expressed in lung cancer. In addition, it implied that the immune system was activated after the operation and the immune block was unarmed, which is a special discovery in our study. Quantitative comparative analysis using mRNA expression data from TCGA database on LUAD demonstrated that there is a significant difference in the expression of ICOS and ICOSL between normal tissues and tumor tissues.^25^ Thus, the expression of ICOS and ICOSL in tumor tissues is relatively special and deserves more focus. Also using immunohistochemical analysis, a previous study evaluated the expression level of immune checkpoint markers in adenocarcinoma (*n* = 142), which involves ICOS. Its result showed that the median value of ICOS expression was 48.77%, and the higher positive expression above the median level was 38%,^26^ which was similar to our study. Therefore, it suggested that ICOS and ICOSL could act as immune monitor indicators of biomarkers in LUAD.

In our study, the expression level of ICOS is related to tumor size, regional nodal status, different stages, and recurrence status. Furthermore, the high expression level of ICOS is concentrated in stage I patients with small tumors, negative regional lymph nodes, and no recurrence. A previous report on lung cancer also showed that the expression of ICOS is related to tumor size.^26^ Another study on draining lymph nodes of lung cancer found that the expression of ICOS in lymph nodes decreased.^27^ The data provided by TCGA show that the mRNA transcription level dynamically fluctuates in stages I–IV of LUAD. Regarding ICOSL, literature studies revealed that its high expression is associated with tumor progression in a variety of cancers such as breast cancer and colon cancer.^9^ An in vitro study showed that ICOSL was obviously concentrated in the lung metastasis site.^28^ However, there are a few earlier reports on ICOSL in lung cancer. The data provided by TCGA on the mRNA transcription of ICOSL exhibited various levels in different stages of lung cancer.^25^ Our study shows that the protein level of ICOSL detected by IHC is related to the clinical stage, which is consistent with information from TCGA. Because clinical tumor staging includes tumor size and local lymph node condition, it can be concluded that both ICOS and ICOSL expressions are closely associated with stage status.

To our knowledge, clinical staging is an important prognostic factor that affects tumor survival. However, multivariate analysis of the survival of all cases in this study shows that neither is an independent factor affecting the OS. Previous data suggest that the expression status of ICOS and ICOSL is closely associated with the prognosis of various tumors.^29^, ^30^, ^31^, ^32^, ^33^, ^34^ Nevertheless, there are a few earlier studies on ICOS and ICOSL on the prognosis of lung cancer, especially for Chinese people. TCGA data demonstrated that low‐expression ICOS was significantly correlated with poor DFS and OS.^35^ Another study, using TCGA and tumor‐infiltrating immune cells, analyzed the expression profile of multiple co‐stimulatory and inhibitory cells, including ICOS. The results revealed that only the expression of ICOS was positively correlated with the prognosis of patients with LUAD.^36^ In addition, detailed research on ICOSL is rather limited; therefore, we detected ICOSL exactly in our study.

Finally, the outcomes of ICOS are similar to the TCGA database, and its expression is beneficial to tumor treatment, but the results of its ligand are obviously different; the expression levels of ICOSL from TCGA were not related to DFS and OS. Our data showed that the high expression of ICOSL is associated with poor prognosis. Possible reasons behind this difference are as follows: Primarily, radical resection can completely remove the primary tumor focus, relieve the immunosuppression caused by tumor immunity, and improve the immune function of patients. Postoperative peripheral blood data showed that NK cell activity and CD4+/CD8+ were significantly higher than those before operation.^37^ The immune status after operation may affect the expression of ICOS and its ligand. The possible reason is that the immune status of the obtained samples is different from that of the TCGA database. Second, the sample size of this group is small, and the proportion of stage I cases is higher than that in TCGA data, which may affect the results. Third, the follow‐up period is longer in the data provided by TCGA. Therefore, the trend of immunohistochemistry may not be completely consistent with mRNA transcription information.

This study has several limitations. First, because this study is exploratory, the sample size is small. Second, this was a retrospective study, and the conditions were often limited. Third, in the case of selection, because of the lack of case selection in terms of time, a group of patients in stage I was too limited for a certain period, with uneven distribution of cases. Individual deviations occur, which affected the results. Fourth, the effect of carbon deposition should be removed. Despite these limitations, this study is the first to characterize the clinicopathologic features and evaluate the relationship between the expressions of ICOS and ICOSL and prognosis in a sample of Chinese patients with LUAD.

In summary, our data showed the expression status of ICOS and its ligand in surgically resected LUAD specimens and clinicopathologic features. We analyzed the correlations between ICOS/ICOSL and prognosis and reached preliminary conclusions; ICOS expression may suggest slower tumor proliferation and better prognosis, which is different from existing research stating that ICOS is harmful to tumor immune in other cancer types. Contrary to this, the high expression of ICOSL may indicate worse tumor development, which needs further investigation and confirmation in a larger sample group. ICOS or ICOSL may be a potential biomarker of lung cancer.

## AUTHOR CONTRIBUTIONS

Research idea and design: Sen Zhang; data acquisition: Xiao‐Kai Zhan; data analysis/interpretation: Xi‐Kun Liu; manuscript drafting and revising: Hong Chen. All authors accept accountability for the overall work in ensuring that questions pertaining to the accuracy or integrity of any portion of the work are appropriately investigated and resolved. The authors read and approved the final manuscript.

## FUNDING INFORMATION

We appreciate the funding from the Chinese Academy of Medical Sciences Innovation Fund for Medical Sciences (CIFMS, nos: 2022‐I2M‐JB‐011, 2022‐I2M‐1‐014), the National Natural Science Foundation of China (82293684), and the National Key R&D Program of China (2022YFA0806400).

## CONFLICT OF INTEREST STATEMENT

All the authors declare that there are no author conflicts and financial conflicts, and all agree with manuscript submission. Sen Zhang is an editorial board member of *AMEM* and a coauthor of this article. To minimize bias, he was excluded from all editorial decision making related to the acceptance of this article for publication.

## ETHICS STATEMENT

This study was approved by the Ethics Committee of Chaoyang Hospital.

## CONSENT FOR PUBLICATION

All the authors have agreed to the publication of this article.

## PATIENT CONSENT FOR PUBLICATION

This study did not involve patients' consent for publication.

## Data Availability

All the data in this study are available based on requirement.
